# Prevalence of burnout and its correlation with resilience among healthcare professionals in Morocco

**DOI:** 10.1192/j.eurpsy.2024.1254

**Published:** 2024-08-27

**Authors:** H. Guider, A. El Alaiki, W. Fadil, H. Hami, F. Hadrya

**Affiliations:** ^1^Laboratory of Biology and Health, Faculty of Sciences, Ibn Tofail University, Kenitra; ^2^University Hassan First of Settat, Higher Institute of Health Sciences, Health Sciences and Technologies Laboratory, Settat, Morocco

## Abstract

**Introduction:**

Burnout syndrome arises as a result of chronic workplace stress that has either been inadequately managed or entirely unaddressed, leading to symptomatic manifestations of emotional exhaustion (EE), depersonalization (DP), and a decreased sense of professional accomplishment (PA).

**Objectives:**

This study evaluated the prevalence of burnout and investigated its correlation with resilience among healthcare professionals in Morocco.

**Methods:**

A self-administered questionnaire survey was conducted in April 2023, comprehensively using the Connor-Davidson Resilience Scale (CD-RISC) and the Maslach Burnout Inventory (MBI) among 296 healthcare professionals stationed across three institutions located in the Casablanca-Settat region.

**Results:**

A total of 158 responses were obtained. Surprisingly, the results indicate that EE was highly prevalent, impacting 43.7% of respondents, while DP was notably affected 44.9% of participants. Conversely, PA was diminished in 58.2% of the respondents. It is worth noting that 44.3% of the participants displayed reduced levels of resilience. Furthermore, statistically significant correlations were observed between resilience and all three dimensions of burnout. Upon gender stratification, the analysis showed that resilience was significantly associated with two burnout dimensions, EE and PA, among male respondents, whereas among their female counterparts, resilience demonstrated a noteworthy correlation with all three dimensions of burnout.

**Conclusions:**

These findings emphasize the pervasive nature of burnout among healthcare professionals and highlight deficiencies in resilience. It is crucial to consider these factors when crafting healthcare policies and devising focused approaches to effectively prevent and manage burnout.

**Disclosure of Interest:**

None Declared

